# Cost-effective and multifunctional acquisition system for *in vitro* electrophysiological investigations with multi-electrode arrays

**DOI:** 10.1371/journal.pone.0214017

**Published:** 2019-03-25

**Authors:** Leonardo D. Garma, Laura Matino, Giovanni Melle, Fabio Moia, Francesco De Angelis, Francesca Santoro, Michele Dipalo

**Affiliations:** 1 Center for Advanced Biomaterials for Healthcare, Istituto Italiano di Tecnologia, Naples, Italy; 2 Dipartimento di Ingegneria Chimica, dei Materiali e della Produzione Industriale. DICMAPI, University of Naples Federico II, Naples, Italy; 3 Istituto Italiano di Tecnologia, Genoa, Italy; 4 Dipartimento di Informatica, Bioingegneria, Robotica e Ingegneria dei Sistemi. DIBRIS, Università degli Studi di Genova, Genova, Italy; Albert-Ludwigs-Universitat Freiburg, GERMANY

## Abstract

*In vitro* multi-electrode array (MEA) technology is nowadays involved in a wide range of applications beyond neuroscience, such as cardiac electrophysiology and bio-interface studies. However, the cost of commercially available acquisition systems severely limits its adoption outside specialized laboratories with high budget capabilities. Thus, the availability of low-cost methods to acquire signals from MEAs is important to allow research labs worldwide to exploit this technology for an ever-expanding pool of experiments independently from their economic possibilities. Here, we provide a comprehensive toolset to assemble a multifunctional *in vitro* MEA acquisition system with a total cost 80% lower than standard commercial solutions. We demonstrate the capabilities of this acquisition system by employing it to i) characterize commercial MEA devices by means of electrical impedance measurements ii) record activity from cultures of HL-1 cells extracellularly, and iii) electroporate HL-1 cells through nanostructured MEAs and record intracellular signals.

## Introduction

Conventionally, electrophysiological recording of *in vitro* cell cultures has been the preferred method for investigating neurons in terms of synaptic activity and ion channel currents [1,2]. In particular, multi-electrode arrays (MEA) have been extensively used for characterizing information processing and computation in complex neuronal networks cultured *in vitro* on the millimeter scale [3].

However, in recent years, MEA recording platforms are becoming also fundamental tools in several research fields other than neuroscience. In fact, electrophysiological recordings of cardiac cells with MEAs are gaining strong interest from the drug discovery and development community[4–6], which exploits this technique to characterize drug effects *in vitro*. For instance, the Comprehensive In vitro Proarrhythmia Assay (CiPA) initiative, promoted by the major global drug administration agencies, has indicated the MEA technology as a promising tool for assessing cardiac drug safety risks[7]. Moreover, MEA systems can be used further as a tool for evaluating the interaction between cells and new bio-interfaces, such as 3D nanostructures or polymeric materials[8,9]. Here, the spontaneous electrical activity of cells is used as parameter to evaluate variations of cell behavior on different materials, morphologies and geometries[10–12]. Lastly, MEA recording is being also combined with other characterization techniques, such as Raman spectroscopy or optical detection, to integrate complementary biological information acquired at different space-time scales[13,14].

Consequently, MEA recording systems are becoming standard equipment for several different research labs active on various bio-related topics not connected directly to neuroscience. However, typical commercial MEA acquisition systems are commercialized at prices in the range of few tens of thousands of euros [15]. The price increases considerably if additional options, such as electrical stimulation, need to be added to the system. In the past 10 years, few approaches have been proposed for low-cost custom systems designed for signal acquisition from electrogenic cells[15–17], with some examples reaching the commercialization[18]. However, these solutions are mainly intended for *in vivo* applications and do not provide a complete platform for *in vitro* electrophysiological recordings using standard *in vitro* MEA devices. For example, the design proposed by J. Rolston et al.[16] requires the addition of a commercial preamplifier board to work for *in vitro* applications. A low-cost acquisition system adapted for *in vitro* applications would thus significantly help to spread the use of MEA technology by enabling *in vitro* electrophysiology measurements to a wider pool of research labs. Furthermore, combined with novel, cost-effective techniques for the fabrication of MEA devices[19,20], such a system would turn *in vitro* electrophysiology into a technique affordable enough to be a standard tool in the analysis of various electrogenic cell cultures for diverse applications.

The recent spread of cost-effective microcontroller/FPGA-based electronics enabled also the design of very-low-cost, fully custom *in vitro* systems[21]. This kind of MEA acquisition platforms is built by acquiring only elemental electronic components and by designing the complete electrical circuits for signal detection, filtering, digital conversion, multiplexing and acquisition. The total cost can be extremely low, in the range of approx. 1,000 €. However, such fully custom systems are considerably difficult to reproduce without expertise in analog/digital electronics, system design and in low-level programming. Thus, although affordable, they are not easy to implement or reproduce.

Here, we provide a solution by presenting the design, the implementation and the experimental characterization of a low-cost MEA acquisition platform that can be assembled directly following the technical designs and software algorithms provided in this work. We show that the system is able to record spontaneous activity with large amplitudes and high signal-to-noise ratio from cultures of the HL-1 cell line grown on both commercial and custom-made MEA biosensors. Moreover, we show that the system can be used also to perform electroporation and to record intracellular action potentials with no need for external stimulators. The complete acquisition system can be assembled with a budget below 6,000€, which is almost an order of magnitude lower than commercial MEA acquisition systems.

## Materials and methods

### Impedance measurements

The system was used to perform impedance measurements on a commercial 64-electrode MEA from Multi Channel Systems GmbH. The measurements were performed using the RHD2132 chips both to generate an AC current waveform and to measure the response of the MEA electrodes. A platinum wire was used as reference electrode to perform a 2-electrode measurement. In order to calibrate the system, the impedance of resistors of 1, 10 and 100 kOhm was measured at the target frequencies of 100, 1000 and 7500 Hz (Fig. B in S1 File). The values obtained from the resistors were used to build a calibration curve for each frequency by means of linear regression (R>0.98 in all cases). When the impedance was measured on the 64 electrodes of the MEA, these calibration curves were used to interpolate the actual impedance based on the value indicated by the system.

### Extracellular recordings

MEAs with 60 planar gold electrodes (60EcoMEA-gr) manufactured by Multi Channel Systems GmbH were used to acquire extracellular signals from HL-1 Cardiac Muscle Cell Line cells.

The chips were cleaned using de-ionized water and sterilized under UV light for 30 minutes. Then the chip surface was coated with a water solution containing 1% fibronectin and 0.02% gelatin and incubated at 37 °C for 2 hours to promote cell adhesion.

HL-1 cells (Sigma-Aldrich) were cultured in supplemented Claycomb medium according to manufacturer’s instructions and passaged three times before plating. On the third passage, 300000 cells were plated on each chip by depositing a single drop in its center, attempting to cover only the area with electrodes. After 10 minutes, 1.5 mL of medium was added. The chips were kept in an incubator at 37 °C and the media was replaced every 24 hours. After 3 days in vitro (DIV), electrophysiological recordings were done every day 30 minutes after the media was replaced. The Peltier cell placed under the MEA was supplied with 2V (I ≈ 0.7A) by a linear power supplier (TT*i*, model QL564) to maintain the temperature at 37 °C during the recordings. The recordings were continued until 6 DIV.

In all cases, the signals were acquired with the following parameters: amplifier bandwidth = 1–10000 Hz, sampling rate = 20 kSamples/s, high pass filter frequency = 5 Hz.

### Electroporation

The LabView libraries provided by INTAN Technologies to control the RHD2000-EVAL board were used to develop a small application to drive the analog output channels on the board. The application generates sequences of monophasic square pulses with a pulse width of 1 ms and arbitrary amplitudes (up to 3.3 V). Connecting the analog output from the board to the connector on the MCS comb, the sequence of pulses could be delivered directly to the electrodes on the MEAs. The custom-made acquisition board provides manual switches to disconnect the MEA electrodes from the INTAN amplifier chips independently, so that the applied electrical stimuli do not damage the amplifier chain.

Custom MEAs with gold nanopillars[22] were used to deliver the electrical stimulation and obtain intracellular recordings from HL-1 cells. The chips cleaning, sterilization, coating and cell plating were described in detail above. After 3 DIV the activity of cells was monitored and stimulation pulses were delivered to the electrodes where spontaneous extracellular action potentials of at least 100 μV were observed. The stimulation pattern consisted of a 1 second-long sequence of 1 ms pulses (1 ms pulse width, 50% duty cycle) and an amplitude of 2 V.

The electrodes on the chip were disconnected from the amplifiers during the stimulation to prevent the stimulation pulses from damaging the amplifiers. Thus, no acquisition was done while the pulses were delivered. After the stimulation, the targeted electrode was manually grounded to discharge any possible charge build-up, all the electrodes were reconnected to the amplifiers and the acquisition was restarted.

The electrical stimulation was delivered sequentially to different electrodes, monitoring its effects for no less than 120 seconds and allowing 10 minutes between repeating the procedure on the same electrode.

A LabView compiled application for performing electroporation by means of the analog output ports of the RHD2000EVAL board is provided as supporting material (S4 File). To use the application, the LabVIEW Run-Time Engine 2017 - (64-bit) must be installed on the computer. This software is freely available from LabView at the link:

http://www.ni.com/download/labview-run-time-engine-2017/6821/en/.

The LabView application allows for selecting the amplitude and the offset of the applied pulses and the duration of the electroporation process. It also provides a graphical preview of the applied pulse configuration (S1 Fig).

### Data analysis

The recordings were imported into MATLAB using the m-code function provided by INTAN Technologies for this purpose. This function is freely available at http://www.intantech.com/files/RHD2000_MATLAB_functions_v2_01.zip. A more complete MATLAB Toolbox for controlling the RHD2000 board is available at http://www.intantech.com/RHD2000_matlab_toolbox.html.

Spikes were detected applying a simple threshold-crossing criterion. The threshold was set on each channel as 4 times the standard deviation. The standard deviation of each channel was computed in all cases for all the signals acquired during a recording session (>1 minute in all cases). To examine the distribution of spike amplitudes and peak frequencies on each electrode, additional thresholds were set to remove artifacts. To this end, all detected spikes with an amplitude smaller than -80 μV or larger than -400 μV were discarded, and only electrodes with more than 20 spikes/second were considered for further analysis. MATLAB functions for importing data and perform spike detection and analysis are provided online at https://github.com/leo-gg/INTAN.

The signal-to-noise ratio was estimated for each electrode based on the waveforms of the detected spikes. First, the waveforms were aligned in time by minimizing their cross-correlation. Then the AP signal was estimated as the mean waveform of the detected APs. The noise was estimated by subtracting the average of the time-aligned waveforms (that is, the estimated AP signal) from each of the detected APs. The SNR was then computed as:

$$SNR_{db}=10{log}_{10}[{\left(\frac{A_{AP\;signal}}{A_{noise}}\right)}^{2}]$$

Where A_AP signal_ is the root mean squared (RMS) amplitude of the estimated AP signal and A_noise_ is the RMS amplitude of the estimated noise.

Unless otherwise noted, all chemicals were acquired from Sigma-Aldrich, USA.

## Results

### Acquisition system

We designed a PCB board that is mechanically compatible with standard commercial 60-channel MEA devices. The PCB board can host two INTAN amplifier boards RHD2132, which are connected to an INTAN RHD2000-EVAL board, which is in turn connected to a PC workstation.

The RHD2132 amplifier boards are 32-channel amplifiers with integrated ADC, 30 ksamples/s max sampling rate per channel while acquiring from all 32 channels, fixed gain of 200 and adjustable filtering. The sampling rate is lower than that of typical commercial systems, which can reach values up to 50 kSamples/s. However, sampling rates in the order of 30 ksamples/s are well beyond the requirements for suitable electrophysiological analysis of action potentials from neurons and cardiomyocytes. The recording vertical resolution of the RHD2132 amplifiers is 0.195 μV. For comparison, commercial *in vitro* systems offer higher vertical resolution down to <1 nV, mainly due to the use of 24-bit Analog to Digital Converters (ADCs) in place of the 16-bit ADCs used by INTAN. By integrating two RHD2132 amplifiers, the board is capable of recording from the 60 channels of commercial MEAs. The RHD2000-EVAL board is an FPGA-based platform that acquires data from the RHD2132 amplifiers and transfers it to a PC *via* USB where it is acquired through a dedicated open-source software (complete with a graphical user interface) provided by INTAN Technologies.

The RHD2000-EVAL board provides additional digital and analog inputs/outputs. Those connections can be used by means of the LabView libraries provided by INTAN Technologies. In particular, the analog outputs can supply voltages between -3.3 V and 3.3 V, and are thus ideal for applying electroporation protocols to electrogenic cells cultured on MEAs with nanopillars as reported in former studies[22,23]. The setup specifications are summarized in Table 1.

**Table 1:** Setup specifications.

Low-cost acquisition system specifications	
**Recording units**	60 channels
**Max sampling rate** **(32-channel recording)**	30 kSamples/s
**Electroporation**	Integrated on 30 channels
**Bandwidths range**	Lower range: 0.1–500 Hz Upper range: 100–15000 Hz
**Nominal INTAN amplifier noise**	2.4 μV_rms_
**Peak-to-peak noise amplitude**	33.79 μV (σ = 12.23,N = 64)
**INTAN Amplifier Crosstalk**	-68 dB (f = 0.1 to 10 kHz)
**Vertical resolution (Voltage Step Size of ADC for Amplifier Input)**	0.195 μV
**Vertical stimulation resolution**	0.1 μV
**Temperature control**	Socket for Peltier cell
**Total cost**	Approximately 6’000€

**Fig 1:**
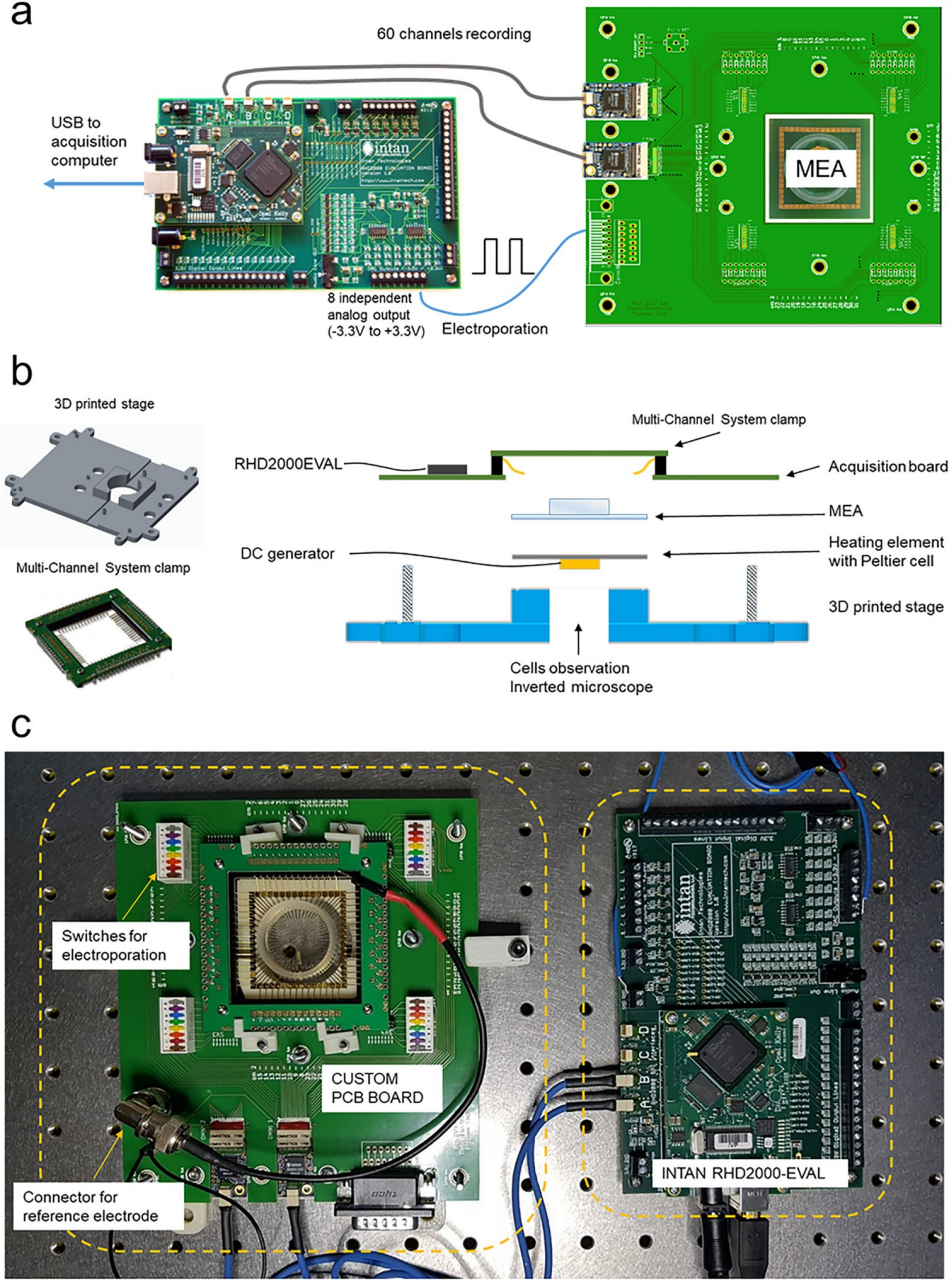
Overview of the setup. a) Connections between the custom PCB board and the INTAN RHD200-EVAL board, showing the electroporation, the acquisition and the computer transfer paths. b) Sketches in the center show all the components of the custom PCB board and the connections to external devices. c) Photographs depict the complete setup.

From the mechanical point of view, the custom-made PCB board interfaces with two parts: a gold spring contact comb from Multi Channel Systems (MCS) GmbH (GSCC1060-Up) and a 3D printed stage. The comb provides spring gold contacts for direct connection with MEA devices. It is mounted and fixed onto the custom-made PCB board (Fig 1 panel B). The 3D printed stage is designed to host the MEA device and to match the PCB board, which is pressed on top of it by screws for pushing the spring gold contacts against the MEA pads. The MEA socket placed on the 3D printed stage is designed with a through-hole space with a diameter of 30 mm (Fig 1 panel B). This slot can be used for observing cells on microscopes or, for instance, for hosting heating elements for warming up the cell cultures during measurements. In the configuration presented in this work, we used a Peltier cell (Laird Technologies, model CP0.8-31-06L) fixed to the bottom of an aluminum plate with a size suitable to host a standard 60-channel MEA from MCS. The aluminum plate can be inserted between the 3D printed stage and the MEA biosensor to maintain the temperature of the cell culture (right sketch in panel B of Fig 1).

The full list of materials and the PCB design are provided in the supporting information S1 File. CAD files of the PCB layout (GERBER format) and of the 3D printed parts (STL) for the support structure and hooks are provided in S2 and S3 Files.

The total cost of an INTAN-based *in vitro* MEA system could be further reduced by replacing the RHD2132 chips with the RHA2132 amplifiers, which offer 32 channels without DAC conversion and multiplexed on a single analog output[24]. With the RHA2132 chips, the RHD2000-EVAL board can be replaced by a PC-based signal acquisition board, such as the DAQ modules from National Instruments. Such configuration may lower the total costs down to approx. 3,000€. However, the system offers limited functionalities, as it does not provide programmable filtering nor impedance measurement. Moreover, it requires the programming of a full data acquisition software and user interface.

### Electrical characterization

**Fig 2:**
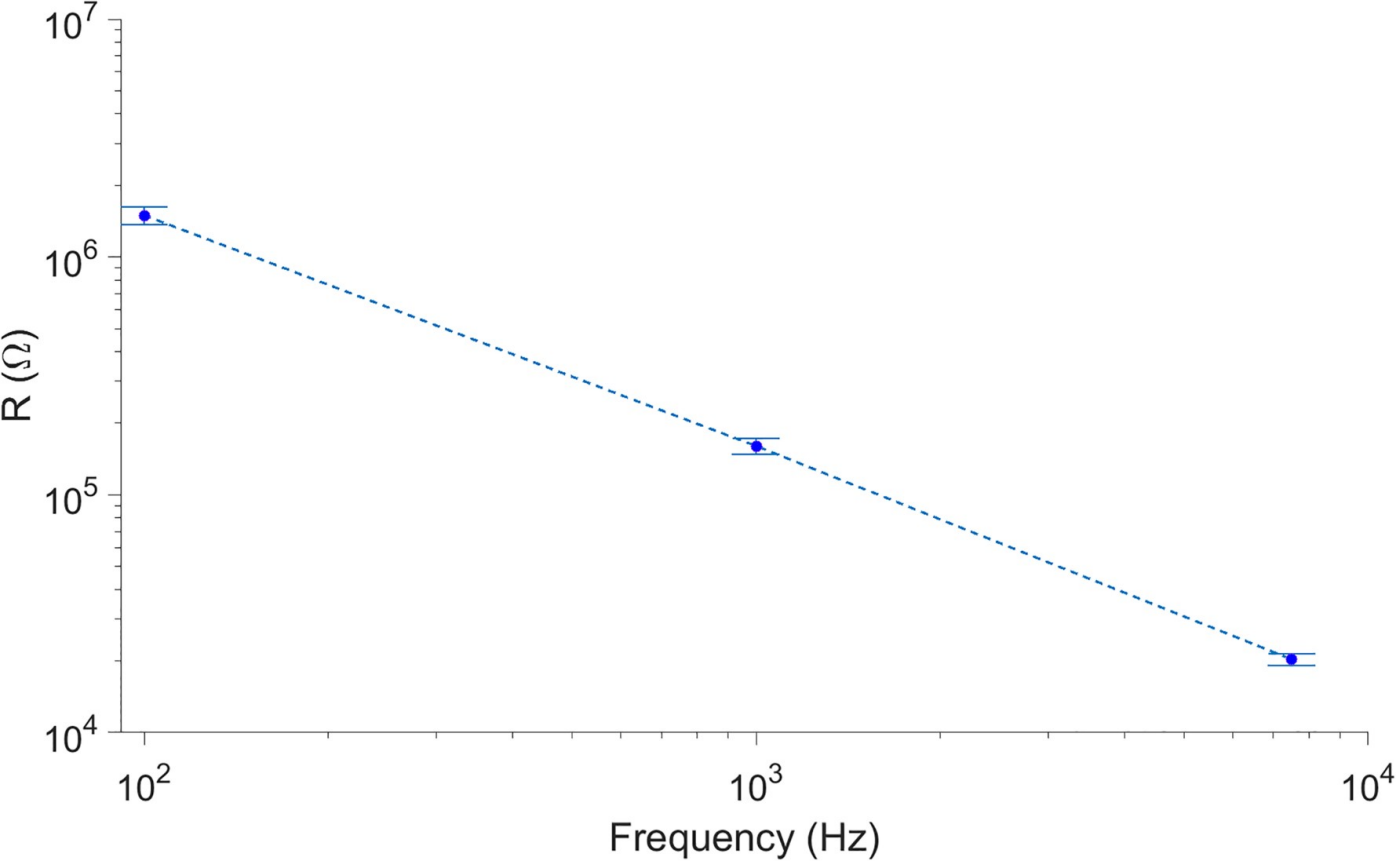
Impedance of gold electrodes from commercial MEA chips. The impedances were measured with the setup at fixed frequencies of 100, 1000 and 7500 Hz. The mean values of the impedance measured at each frequency are shown as blue dots connected by a discontinuous line. The values of the standard deviations are indicated by the error bars.

We used the built-in capabilities of the INTAN interface software and the RHD2000-EVAL board to characterize the electrodes of commercial MEAs by measuring their impedance at 100, 1000 and 7500 Hz. The observed mean impedances at 100, 1000 and 7500 Hz were 1235.4 (σ = 124.75), 160.06 (σ = 12.88) and 39.28 (σ = 1.12) kOhm, respectively (N = 64). The observed log-linear behavior is typical of gold electrodes in planar MEAs[25]. The measurements were very consistent, with deviations smaller than 8.5% of the mean value in all cases (Fig 2). The impedance values observed at 1 kHz are approximately in the range of those reported by the manufacturer for pristine chips (100 kOhm)[26] and in agreement with the value measured with a commercial potentiostat (119.42 kOhm, at 1 kHz σ = 5.58 for N = 5, Fig. C in S1 File).

### Electrophysiological recordings

**Fig 3:**
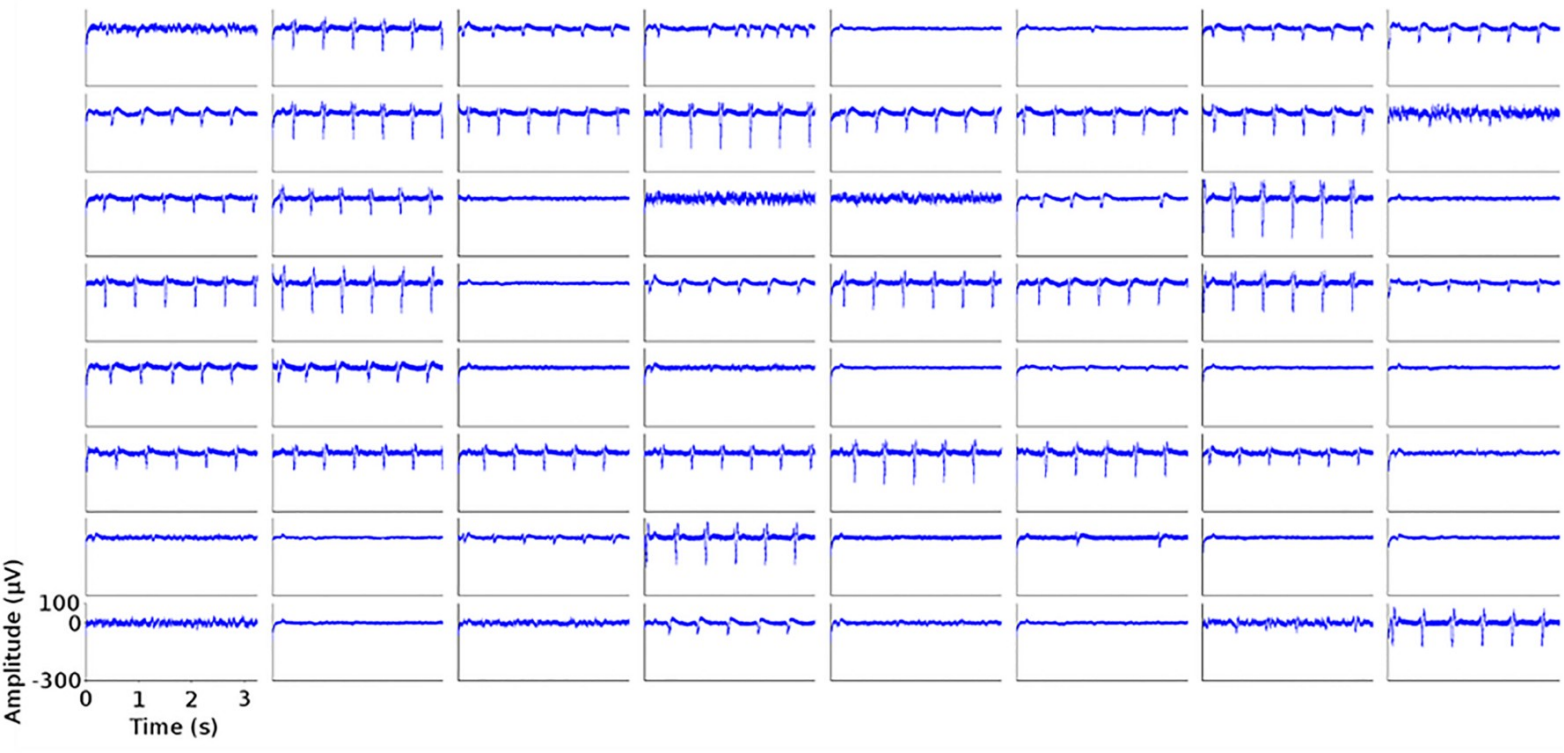
Sample traces acquired from a commercial MEA chip. Each panel shows a 3-second long voltage trace acquired from a different channel.

We used commercial MEAs with gold electrodes to acquire extracellular signals from cardiomyocytes-like (HL-1) cultures. During each recording, a number of electrodes presented typical signals corresponding to action potentials whereas the rest captured only background noise (Fig 3). Action potentials (APs) were detected using a threshold-crossing criterion to detect action implemented in MATLAB.

**Fig 4:**
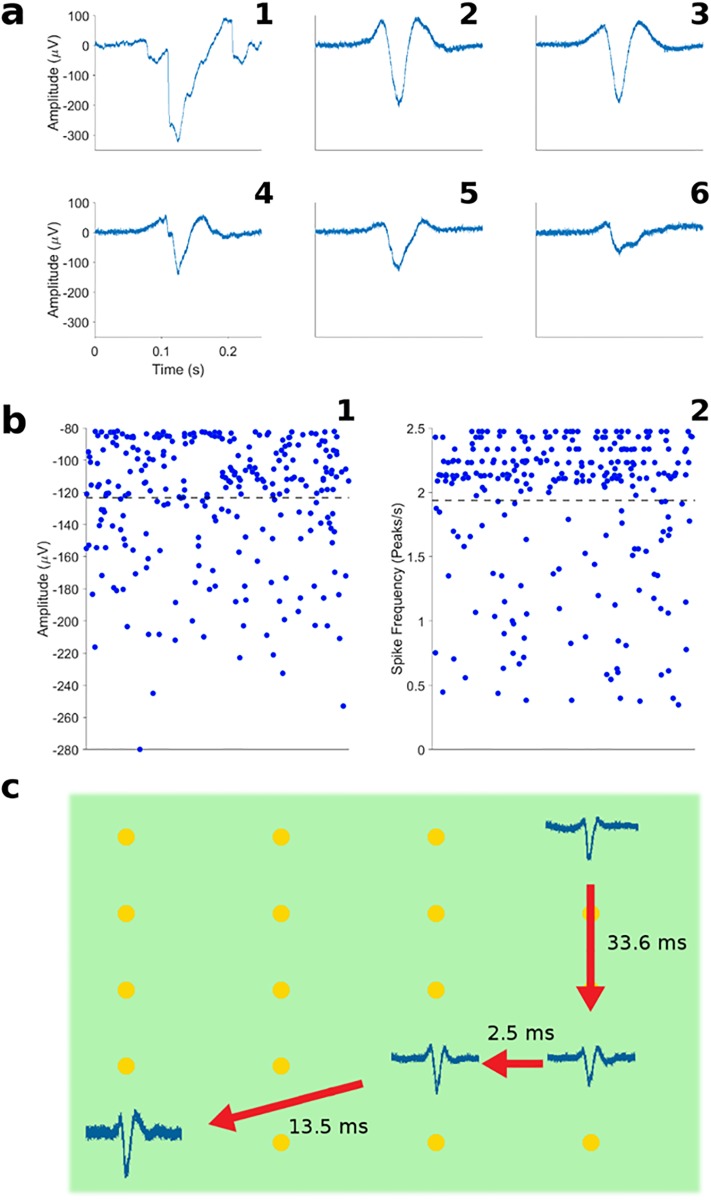
Extracellular signals. a) Waveforms of detected spikes with different amplitudes. b) 1. Distribution of the average spike amplitudes per electrode. 2. Distribution of spike frequencies per electrode. c) Propagation of sequential APs across the surface of an MEA chip. The detected spikes were placed on the 2D location of the electrodes where they were detected. In the image, four spikes detected sequentially in time are shown in their location on the chip surface. The direction of the arrows indicates the temporal order of the spikes, with the time between APs annotated next to the arrows.

Most of the detected APs presented waveforms, with a positive peak (<100 μV) followed by a negative downwards peak (80 to 300 μV) and a positive hump (<100 μV) (Fig 4a, panels ii-v). Some APs with lower amplitudes exhibited a wide plateau after the negative peak and missed the positive hump at the end of the wave (Fig 4a, panel vi). These observations are in agreement with typical HL-1 extracellular potentials described in the literature[27–29]. Only in few instances (0.1%), APs with large amplitudes exhibited distorted waveforms (Fig 4a, panel i). These distorted waveforms were registered in three different channels in which no other spikes were otherwise observed. These waveforms appeared with a period of 800 ms in the first 30 seconds of the recording; they were not observed again afterwards. Thus, we find it reasonable to attribute them to a transient artifact rather than to an intrinsic instability of the system. Panel i in Fig 4b shows the per-electrode average amplitude of peaks detected on 269 electrodes across 6 different commercial MEAs. The plot shows the amplitude value for each individual electrode. The values are spread randomly along the horizontal axis to increase readability. Panel ii depicts the per-electrode average frequency of AP occurrences on the same MEAs. On average (N = 269 electrodes), 1.94 spikes per second (σ = 0.57) with an amplitude of –123.4 μV (σ = 39.12) were detected on each electrode. The estimated signal-to-noise ratio ranged from 8.9 to 17.6 dB, depending on the amplitude of the peaks detected on each electrode.

Using the layout of the electrodes on the MEA, we mapped each detected AP to its location on the surface of the chip. In this way, it was possible to track the propagation of action potentials in space across the cell culture (Fig 4c). With an electrode diameter of 100 μm and an inter-electrode spacing of 700 μm, the observed propagation velocities of AP events (considering the threshold-crossing times as the events timestamps) were in the range of 0.047 to 0.32 m/s. These observations are consistent with previous propagation velocities observed in cardiomyocytes[30].

**Fig 5:**
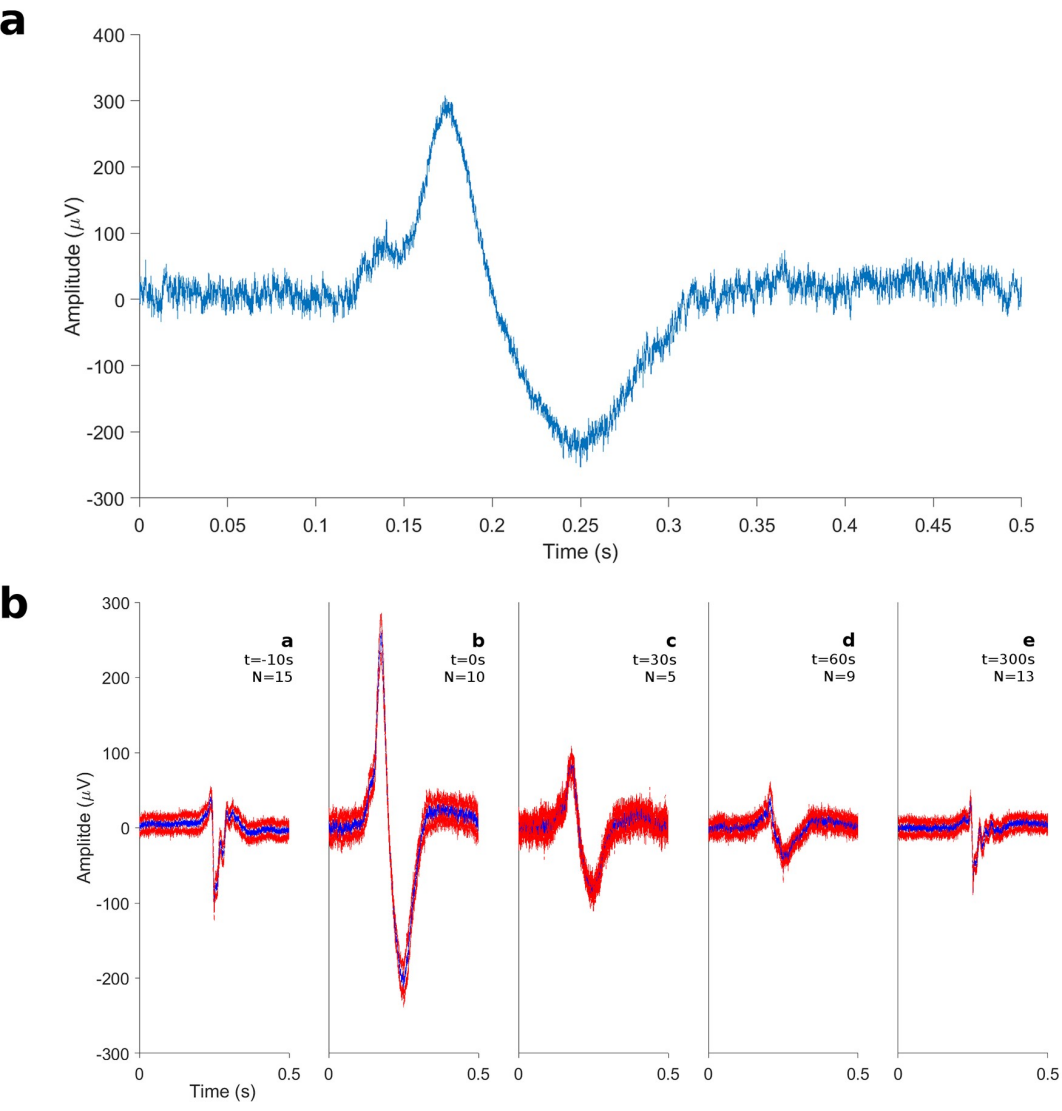
Intracellular signals. a) Typical intracellular waveform observed immediately after applying the electroporation protocol. b) Average spikes detected before and at different times after electroporation. Each panel shows the average of all the spikes detected in the 10 seconds after the indicated time. The average waveform is shown in blue whereas the mean plus or minus the standard deviation on each point is shown in red to indicate the magnitude of the uncertainty. The number of spikes used to obtain the average is indicated on each panel.

We took advantage of the capabilities of the RHD2000-EVAL board to deliver voltage pulses with tunable amplitude to stimulate HL-1 cells. For this application, we used custom MEAs with gold nanopillars[13] (the LabView application for delivering the electrical stimuli is provided in S4 File and online at https://github.com/leo-gg/INTAN). We applied monophasic 2 V square pulses for 1 second to electrodes where large (< -100μV) extracellular spikes had been initially detected. As shown in Fig 5a, intracellular-like signals could be detected on the same electrodes after the electrical stimulation, implying that the pulses had effectively porated the cell membrane. The shape and amplitudes of the observed intracellular signals is in agreement with the results previously observed on cells electroporated with sharp nanopillar structures[22]. By monitoring the signals on the same electrode, we could observe that the shape and amplitudes of the action potentials returned to the pre-stimulation values after few minutes (Fig 5b), indicating that the re-sealing of the membrane had taken place.

## Conclusion

In this work, we provide a complete set of tools for implementing a low-cost *in vitro* MEA acquisition system and for performing recording of spontaneous activity of electrogenic cells, both extracellular and intracellular by means of integrated electroporation capabilities. We show the recording of activity from HL-1 cell cultures detected extracellularly on commercial MEAs with 60 channels. The detected extracellular action potentials present typical waveforms and amplitudes as reported in the literature. Moreover, using custom MEAs with gold nanopillars, and without any additional hardware, we conducted poration experiments in which we could 1) observe intracellular signals and 2) monitor the process of membrane re-sealing by observing the transition from intracellular signals back to extracellular ones. Additionally, we showed that the system can also be used to obtain impedance measurements to characterize MEA devices. In summary, this evidence demonstrates that the setup is fit to be used as a platform for the study of the activity of cardiac cell cultures for a fraction of the cost of commercially available setups. The total cost for assembling the system is almost an order of magnitude lower than that of commercial setups. Furthermore, open-source software is readily available for interfacing with the setup, and we showcased how its functionalities can be easily accessed and customized through the existing libraries, either for hardware control (LabView) or for data analysis (MATLAB).

## Supporting information

S1 File List of components and impedance measurements. (DOCX)

S2 File PCB layout. (ZIP)

S3 File 3D printed parts. (ZIP)

S4 File LabView program for electroporation. (ZIP)

S1 Fig Screenshot of the LabView electroporation application. (TIF)
