# Supplementary material for: Cost-effective and multifunctional acquisition system for in vitro electrophysiological investigations with multi-electrode arrays
Source: PLoS One. 2019 Mar 25;14(3):e0214017. doi: 10.1371/journal.pone.0214017 (PMC6433224; doi:10.1371/journal.pone.0214017)
Supplement: S1 File — (DOCX) [file pone.0214017.s001.docx]

**S1 - List of components**

The custom PCB board hosting the INTAN amplifiers has been fabricated on a two-layers FR4 standard PCB with a thickness of 1.6 mm. We avoided thinner FR4 materials to maintain high robustness necessary to press the contact springs of the Multi Channel System clamp onto the MEA pads. Figure A shows the top and the bottom sides of the layout of the custom PCB board. All layers’ designs are available as supporting material for reproducing the board.

***
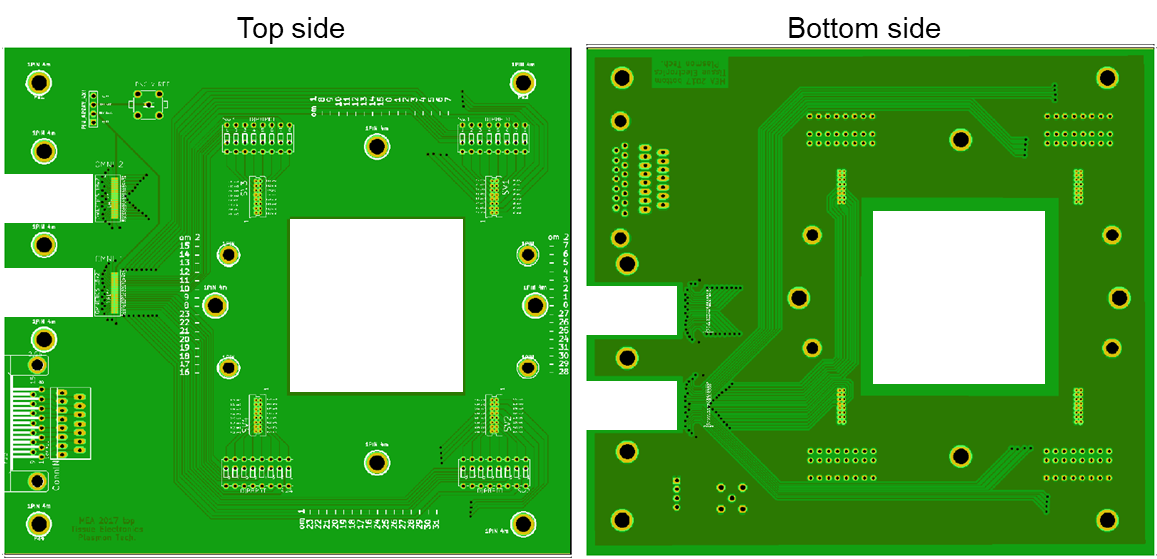
***

**Figure A Custom PCB layout**

**Table A List of setup components and prices**

| Component | Amount | Description | Price |
| --- | --- | --- | --- |
| RHD2000EVAL | 1 | FPGA based board for acquiring signals from INTAN amplifier chips | 2736 EUR (3175 USD) |
| RHD2132 | 2 | INTAN amplifier chip with 32 channels | 1558 EUR (1790 USD) |
| RHD2000 SPI Interface cable | 2 | INTAN cables to connect the amplifiers to the acquisition board | 514 EUR (590 USD) |
| PCB board | 1 | Electronic board that connects to two amplifier chips and to MEA devices. | 500 EUR |
| 3D printed stage | 1 | Mechanical stage for hosting the MEA device | 120 EUR |
| GSCC1060-Up | 1 | MEA clamp with spring contacts for connecting with the MEA pads | 343 EUR |
| 3D printed hook | 2 | Mechanical part for fixing the GSCC1060-Up to the PCB board. | 50 EUR |
|  |  | **Total cost:** | ≈ 5821 EUR |

**Table B PCB board components**

| ID | Reference | Package | No. | Mounting | No. pins | Code | Supplier |
| --- | --- | --- | --- | --- | --- | --- | --- |
| 1 | OMNETICS 18x2,OMNI1,OMNI2 | SSOP24 | 2 | smd | 36 | NPD-18-AA-GS | Omnetics |
| 2 | ConnSt | 22-23-2081 | 1 | Through Hole | 15 | 765-5705 | RS |
| 3 | 1PIN | 1pin | 4 |  | 1 |  |  |
| 4 | SV1,SV2,SV3,SV4 | 16PRECISION | 4 | Through Hole | 16 | 702-0284 | RS |
| 5 | ConnIN | F15HP | 1 | Through Hole | 15 | 495-9726 | RS |
| 6 | Sw1,Sw3,Sw4,Sw2 | DIP08S | 4 | Through Hole | 24 | 321-149 | RS |
| 7 | 1PIN 4m | 1pin4m | 8 |  | 1 |  |  |
| 8 | BNC V REF | B35N61 | 1 | Through Hole | 5 | 546-4027 | RS |
| 9 | PIN_ARRAY_4X1 | PIN_ARRAY_4x1 | 1 | Through Hole | 4 | 251-8115 | RS |

**Impedance measurements**

**
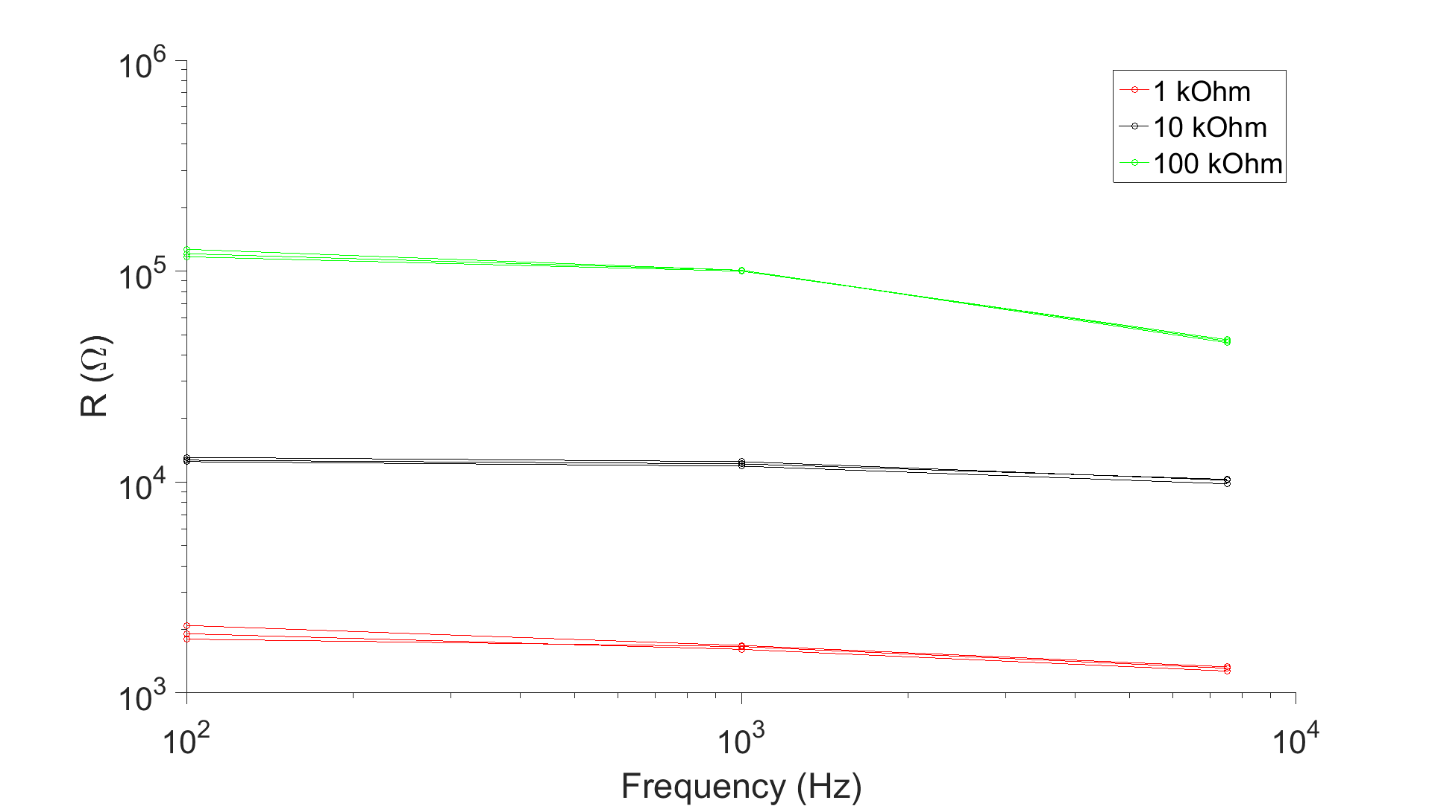
**

**Figure B Impedance measurement of resistors.**

**
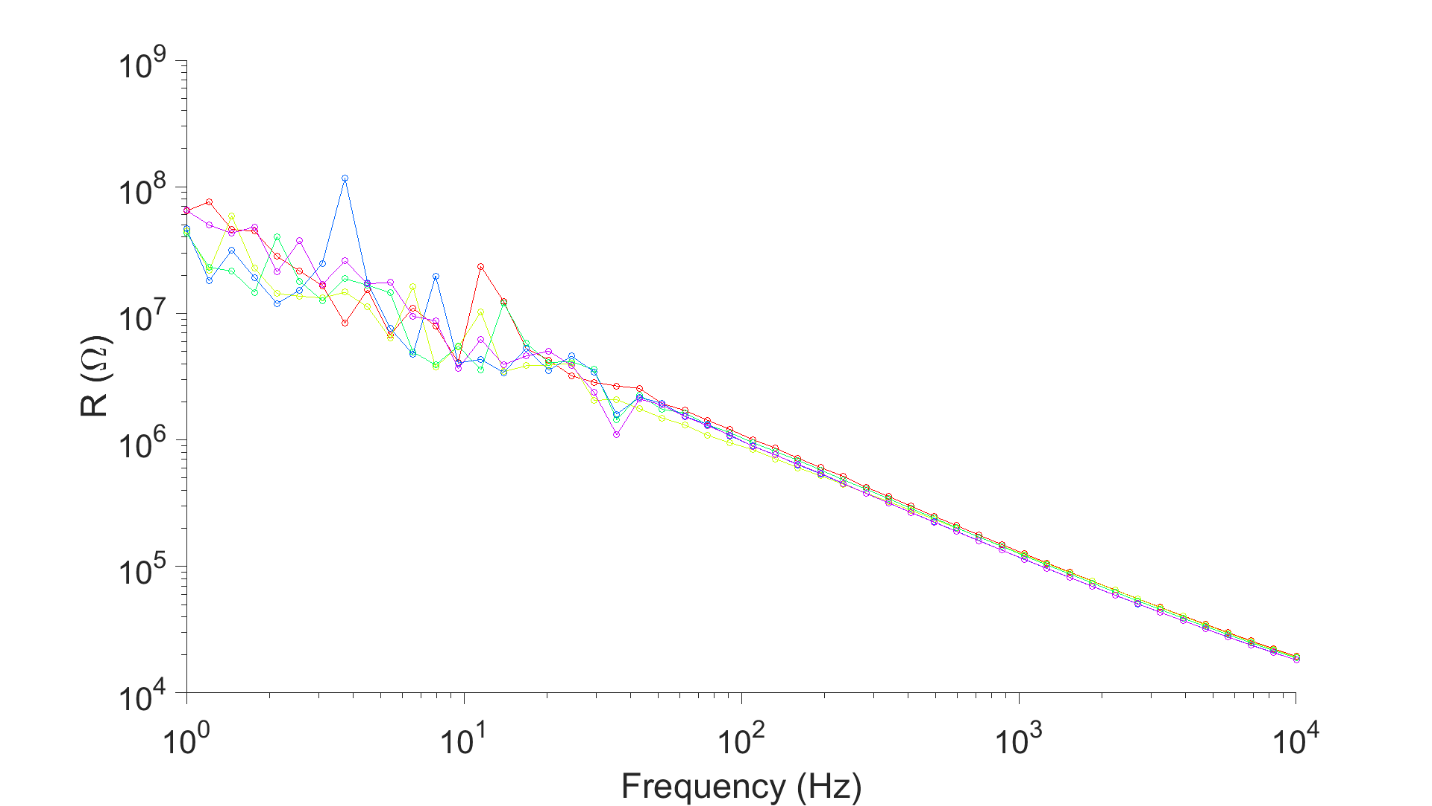
**

**Figure C Impedance spectroscopy of a commercial MEA with 60 gold electrodes obtained with a commercial potentiostat.**
