# Supplementary figures and images for: Cost-effective and multifunctional acquisition system for in vitro electrophysiological investigations with multi-electrode arrays
Source: PLoS One. 2019 Mar 25;14(3):e0214017. doi: 10.1371/journal.pone.0214017 (PMC6433224; doi:10.1371/journal.pone.0214017)

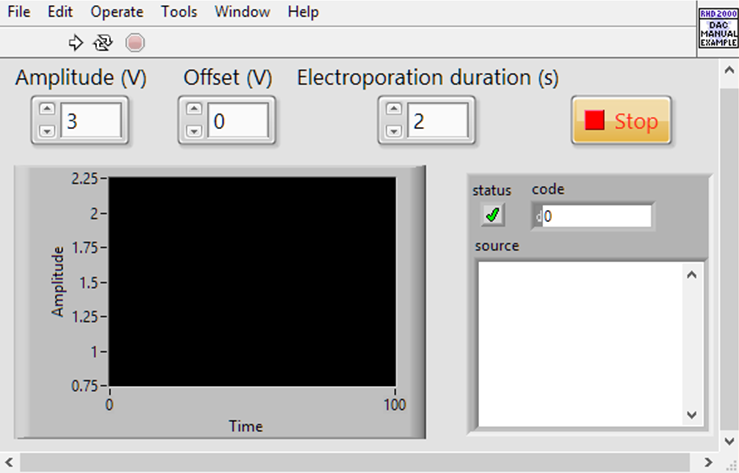

Supplement: S1 Fig — (TIF) [file pone.0214017.s005.tif]
